# Maternal erythrocytosis as a risk factor for small for gestational age at term in high altitude

**DOI:** 10.61622/rbgo/2024rbgo98

**Published:** 2025-01-23

**Authors:** Wilfredo Villamonte-Calanche, Marco Antonio Salazar-Zegarra, Cleto De-la-Torre-Dueñas, Alexandra Villamonte-Jerí, Adaí Vera-Luza, Milagros Hilari Bustinza-Apaza, Nuria Huanca-Huirse

**Affiliations:** 1 Maternal Perinatal Medicine Research Center Research Institute Andina University of Cusco Peru Maternal Perinatal Medicine Research Center, Research Institute, Andina University of Cusco, Peru.; 2 Obstetrics and Gynecology Department Adolfo Guevara Velazco National Hospital of ESSALUD Cusco Peru Obstetrics and Gynecology Department, Adolfo Guevara Velazco National Hospital of ESSALUD, Cusco, Peru.; 3 Academic Department of Mathematics and Statistics National University San Antonio Abad del Cusco Cusco Peru Academic Department of Mathematics and Statistics, National University San Antonio Abad del Cusco, Cusco, Peru.; 4 Occupational Therapy Department Carroll University Waukesha Wisconsin USA Occupational Therapy Department, Carroll University, Waukesha, Wisconsin, USA.; 5 Professional School of Human Medicine Faculty of Health Sciences Andina University of Cusco Cusco Peru Professional School of Human Medicine, Faculty of Health Sciences, Andina University of Cusco, Cusco, Peru.; 6 Professional School of Human Medicine Faculty of Health Sciences San Antonio Abad University of Cusco Cusco Peru Professional School of Human Medicine, Faculty of Health Sciences, San Antonio Abad University of Cusco, Cusco, Peru.

**Keywords:** Pregnancy, Pregnant women, Gestational age, Polycythemia, Altitude, Hypoxia, Fetal growth retardation, Neonatal mortality, Morbidity, Hemoglobin, Small for gestational age, Risk factors

## Abstract

**Objective:**

To determine if maternal erythrocytosis is a risk factor for small-for-gestational age at term at 3,400-m altitude in pregnant women without intercurrent disease.

**Methods:**

Analytical study of retrospective cohorts at Cusco, a city at 3,400-m altitude. Our participants were 224 and 483 pregnant women with and without exposure to maternal erythrocytosis, respectively. A logistic regression with the goodness of fit to the proposed model was also performed with the Hosmer and Lemeshow test, evaluating the small-for-gestational-age results with or without exposure to hemoglobin >14.5 g/dl.

**Results:**

The incidence of small-for-gestational-age was 6.9% for this entire cohort. The maternal erythrocytosis during gestation without any maternal morbidity at 3,400-m altitude has an ORa=0.691 (p=0.271) for small-for-gestational-age at term. Inadequate prenatal control has an ORa=2.115 (p=0.016) for small-for-gestational-age compared to adequate prenatal control.

**Conclusion:**

Maternal erythrocytosis in pregnant women without any morbidity is not a risk factor for small-for-gestational-age at 3,400 m-altitude.

## Introduction

Maternal erythrocytosis (ME) is defined as the presence of hemoglobin (Hb) >14.5 g/dl during pregnancy.^(1)^ ME is a risk factor for preeclampsia, maternal mortality,^(2)^ and small-for-gestational-age (SGA) in high-altitude cities (> 2500 m)^(1)^ when compared with low-altitude cities. Around the world, >80 million people live at high altitudes.^(3)^ SGA is considered as a weight below the 10^th^ percentile for the gestational age. In addition, SGA is a risk factor for death and sickness in the neonatal period.^(4)^ Moreover, SGA is associated with various pathologies in adulthood, such as cardiovascular diseases and diabetes mellitus.^(5)^

SGA has a high prevalence in high-altitude cities due to hypobaric hypoxia (HH),^(6)^ as well as it is related to ME. On the other hand, genetic variations in the genes that regulate oxygen sensitivity, metabolic homeostasis, and vascular influence fetal growth (FG) and birth weight (BW) as part of the process of adaptation to HH of Andean and Tibetan pregnant women.^(7)^ Therefore, the newborn’s weights are similar to those described at sea level without ME, especially in Tibetan women.^(8)^

The Tibetan population has adapted well to HH. However, the Andean population had a different degree of adaptation. For example, Peruvian pregnant women adapted partially to HH depending on the geographical localization. Compared to North Peru, South Peru had better adaptations to HH^(1)^ since the BW at Cusco (3,400 m) is slightly lower than the ones at sea level. On the other hand, the Hb level in the third trimester of pregnancy in Cusco is 13.82 g/dl (95% CI 13.70-13.9).^(9)^ This higher level of Hb increases the frequency of ME in Peruvian cities located >3000 m altitude, such as Cerro de Pasco (4340 m), Huancané (3825 m), and Cusco, where these frequencies are 47.9%, 40, 9%, and 25.7% respectively.^(1)^

The objective of the present study was to determine if ME is a risk factor for SGA in pregnant women without intercurrent disease in high-altitude cities.

## Methods

This was a retrospective cohort study. The medical records of pregnant women who gave birth between February 2020 and January 2021 at the Adolfo Guevara Velazco National Hospital (AGVNH) of ESSALUD (Social Health Insurance) in Cusco, Peru, were used. This tertiary-level hospital is a unique reference for the entire region, where the middle-class population is generally treated.^(10)^

The inclusion criteria for the exposed group were single pregnancy at term by date of last menstrual period (37-42 weeks), a Hb level >14.5 g/dl measured in venous blood in the last trimester of pregnancy (34 ± 2 weeks), without altitude-correction by Sysmex XN-1000-Hematology-Analyzer, absence of any maternal disease, and absence of fetal anomalies. The exclusion criteria for the exposed group were multiple gestations, gestational age <37 weeks, presence of any maternal disease, fetal anomalies, and delivery in a place other than the hospital mentioned above. Both criteria for the unexposed group are the same, except that the Hb level was ≥11 and ≤14.5 g/dl.

Using Epidat 4.2, power calculations determined that 660 women (220 with ME and 440 without ME) were required to achieve 90% power and a confidence level of 95 %, assuming an SGA incidence of 20% in the ME group and 10.5% in the non-ME group.^(11)^

Maternal erythrocytosis was considered the exposure variable. The SGA was the outcome, defined as the BW below the 10th percentile according to the anthropometric table for birth in altitude (TANA).^(12)^ The number of pregnancies was classified as single, 2-4, and 5 or more. The number of previous abortions refers to the number of pregnancies that ended before 20 weeks. Live children were the number of children born alive >20 weeks. The level of education was determined by the highest level of school the participants completed. The participants were classified as illiterate, high schooler, and higher education. The mother’s occupation refers to the work condition for which she receives monthly remuneration; the occupation was classified as unemployed, employed, and self-employed. The socioeconomic condition was based on the participant’s location and classified as wealthy or poor by Peru’s district and provincial monetary poverty map.^(13)^ The ubication of the place of birth was defined as low altitude (<2,500 m) and high altitude (≥2,500 m).^(14)^ Prenatal control was the number of visits to a doctor or midwife to be evaluated. It was classified as absent, inadequate (1-5 visits), and adequate (≥6 visits).^(15)^ None of the pregnant women reported cigarette consumption; therefore, this variable was not considered.

The neonatal anthropometric measurements were weight, height, ponderal index, head circumference (HC), chest circumference, and head-circumference-birth-weight index. The last one resulted from the product of the HC in cm multiplied by 1,000 and divided by the BW in g. The ponderal index is the BW in g by 100 divided by the cube of the height in cm. All these measurements were converted into z-score values using TANA to homogenize the variables.

The information was obtained from the registry of the obstetrics service of the hospital where it was studied. A database was built in the Microsoft Excel program under the supervision of the most experienced researcher. The exposed pregnant woman was selected from the registry above, as well as the following two unexposed pregnant women. The same procedure was carried out until the final study population was obtained.

The database was exported to the IBM SPSS 25 statistical package. The categorical variables were described as absolute and relative frequencies, and the quantitative variables as mean and standard deviation or median and interquartile ranges depending on their distribution. Chi-square or Fisher’s exact statistical tests were used for the bivariate analysis between the categorical variables and the results. For quantitative variables, the t-student test was used if the assumptions were met or, otherwise, the Mann-Whitney U test was used.

To determine whether ME was a risk factor for SGA, a logistic regression model with goodness of fit was used. In the logistic regression model, we included the variables that influenced SGA in the bivariate analysis. We determined crude odds ratios (ORc), and adjusted odds ratios (ORa), 95% confidence intervals (95% CI). p<0.05 was statistically significant.

The research protocol was developed, evaluated, and finally approved by the AGVNH Ethics Committee (Letter No 80-CE-GRACU-ESSALUD-2020). Patient confidentiality was preserved through specific coding.

## Results

**Figure 1:**
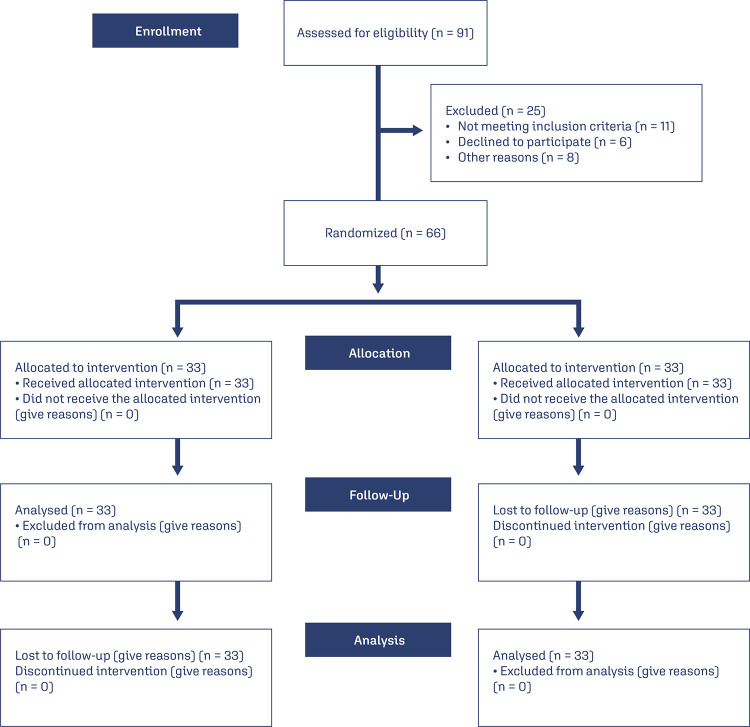
Study flowchart

During the evaluated period, 707 pregnant women were chosen for the study from a total of 2,500, according to the inclusion and exclusion criteria for pregnant women exposed to ME and those not exposed to ME (Figure 1).

The incidence of SGA was 6.9%. 224 and 483 exposed and non-exposed pregnant women were evaluated, obtaining 658 AGA and 49 SGA (19 and 30 in the exposed and non-exposed pregnant women). The variables that showed a significant difference between the SGA and AGA groups were pregestational weight, body mass index, and prenatal control (Table 1).

**Table 1:** Maternal-perinatal characteristics of SGA and AGA neonates

Characteristics	SGA		AGA		p-value
	Median	Interquartile range	Median	Interquartile range	
Maternal					
Age (years)	32.0	27.0 – 36.0	31.0	28.0 – 36.0	0.751
Pregnancies	2.0	1.0 – 3.0	2.0	1.0 – 3.0	0.401
Live births	1.0	0.0 – 2.0	1.0	0.0 – 1.0	0.614
Miscarriages	0.0	0.0 – 1.0	0.0	0.0 – 1.0	0.625
Prenatal care	7.0	4.0 – 8.0	8.0	6.0 – 9.0	0.011
Inicial weight (kg)	57.0	52.8 - 60.5	60.0	55.0 – 67.0	0.010
Weight gain (kg)	11.2	9.0 - 15.8	12.5	10.0 – 16.0	0.389
Height (m)	1.52	1.49 - 1.58	1.53	1.50 - 1.57	0.621
Body mass index	24.2	22.8 - 26.0	25.3	23.3 - 28.4	0.008
Hemoglobin (g/dl)	14.3	13.4 - 14.8	14.0	13.2 - 14.7	0.322
MCV (fl)	88.0	85.8 - 90.7	88.2	85.5 - 90.8	0.999
MCH (pg)	31.0	30.0 - 31.8	30.7	29.5 - 31.7	0.469
MCHC (g/dl)	34.8	34.3 - 35.4	34.6	34.0 - 35.3	0.220
RDW CV (%)	42.5	40.7 - 46.2	43.8	41.9 - 45.8	0.158
RDW SD (fl)	13.3	12.9 - 14.2	13.5	13.1 - 14.3	0.233
Neonatal					
BW (z score)	-2.19	-2.51 to - 1.62	0.09	-0.70 to 0.80	<0.001
Height (z score)	-1.56	-2.60 to -0.60	-0.07	-0.76 to 0.80	<0.001
Ponderal Index (z score)	-1.30	-1.90 to -0.22	0.21	-0.66 to 1.07	<0.001
HC (z score)	-1.45	-2.35 to - 0.38	0.21	-0.58 to 1.08	<0.001
TC (z score)	-1.46	-2.31 to -1.02	0.24	-0.61 to 1.05	<0.001
HC/BW index (z score)	2.45	1.99 to 2.87	0.10	-0.87 to 0.72	<0.001

MCV - mean corpuscular volume; MCH - mean corpuscular hemoglobin; MCHC - mean corpuscular hemoglobin concentration; RDW CV - variation coefficient of red blood cell distribution width; RDW SD - standard deviation of red blood cell distribution width; BW - birth weight; HC - neonatal head circumference; TC - neonatal thoracic circumference; SGA - small for gestational age; AGA - adequate for gestational age

In both groups, most women had higher education, were born in high-altitude cities, and had independent work (Table 2). There were no pregnant women who were illiterate in the population studied.

**Table 2:** Maternal qualitative characteristics of SGA and AGA neonates

Characteristics	SGA	AGA	p-value
	n(%)	n(%)	
Level of instruction			
High schooler	1(0.2)	3(6.3)	0.361
Higher education	655(99.8)	48(93.7)	
Occupation			
Unemployed	12(24.5)	139(21.1)	0.848
Employed	15(30.6)	204(31.0)	
Self-employed	22(44.9)	315(47.9)	
Place of birth			0.940
Low altitude	6(12.2)	83(12.6)	
High altitude	43(87.8)	575(87.4)	
Home district			
Poor	0(0.0)	18(2.7)	0.241
Wealthy	49(100.0)	640(97.3)	

SGA - small for gestational age, AGA - adequate for gestational age

Analyzing the factors through logistic regression for the presence of SGA allowed us to obtain ORc values, where pregestational maternal weight, prenatal control, body mass index, and maternal age influenced the result (p<0.05). By performing the same statistical evaluation with goodness of fit, a model was determined based on the previous factors. It showed that ME does not affect the presence of SGA compared the AGA group. We also obtained that an inadequate prenatal control increased the risk of having an SGA neonate by 231.6% compared to an adequate prenatal control. Similarly, maternal age between 20-35 years has a protective effect for this problem since it reduces the possibility of having an SGA by 80.5% compared to pregnant women <20 years (Table 3). The Hosmer-Lemeshow test was 0.663.

**Table 3:** Crude and adjusted models to evaluate the association between maternal factors and small for gestational age in pregnant at 3400-m altitude

	AGA	SGA	Crude			Adjusted		
	n(%)	n(%)	ORc	CI 95%	p-value	ORa	CI 95%	p-value
Maternal factors								
Hemoglobin 11.0 - 14.5 g/dl	453(93.8)	30(6.2)	Ref			Ref		
Hemoglobin >14.5 g/dl	205(91.5)	19(8.5)	0.715	0.393 - 1.299	0.271	0.691	0.375 - 1.271	0.234
Age								
< 20 years	7(70)	3(30)	Ref			Ref		
20 - 35 years	484(93.8)	32(6.2)	0.154	0.038 - 0.625	0.009	0.195	0.046 - 0.830	0.027
> 35 years	167(92.3)	14(7.7)	0.196	0.046 - 0.841	0.028	0.274	0.060 - 1.252	0.095
Pregnancies								
1	196(92.5)	16(7.5)	Ref					
2 - 4	436(93.8)	29(6.2)	0.815	0.433 - 1.535	0.526			
≥5	26(86.7)	4(13.3)	1.885	0.585 - 6.069	0.288			
Births alives								
None	278(92.4)	23(7.6)	Ref					
1 - 3	374(94)	24(6)	0.776	0.429 - 1.403	0.401			
≥4	6(75.0)	2(25.0)	4.029	0.769 - 21.102	0.099			
Miscarriages			1.081	0.704 - 1.662	0.721			
Grade of Instructions								
High schooler	3(75)	1(25)	Ref					
Higher education	655(93.2)	48(6.8)	0.220	0.022 - 2.154	0.193			
Occupation								
Unemployment	139(92.1)	12(7.9)	Ref					
Self-worker	315(93.5)	22(6.5)	0.809	0.389 - 1.681	0.570			
Employee	204(93.2)	15(6.8)	0.852	0.387 - 1.875	0.690			
Place of Birth								
Sea level	83(93.3)	6(6.7)	Ref					
Altitude	575(93)	43(7)	1.034	0.427 - 2.506	0.94			
Economic conditions								
Wealthy	640(92.9)	49(7.1)	Ref					
Poor	18(100)	0(0)	0.000	0.000				
Prenatal care visit								
≥ 6	507(94.6)	29(5.7)	Ref			Ref		
1 - 5	151(90.9)	20(11.7)	2.316	1.273 - 4.211	0.006	2.115	1.151 - 3.887	0.016
Pregestational weight			0.955	0.922 - 0.990	0.011			
Weight increases during pregnancy			0.976	0.917 - 1.039	0.447			
Height			0.184	0.002 - 22.294	0.489			
Body mass index			0.901	0.825 - 0.924	0.021	0.902	0.823 – 0.989	0.028

AGA - Adequate for gestational age; SGA - small for gestational age; Model adjusted for Maternal age, antenatal visit and body mass index

## Discussion

The ME during gestation without concomitant maternal morbidity at 3,400-m altitude is not a risk factor for SGA at term. This finding differs from previous studies, where this factor has been found to increase the risk of SGA by 1.4 times (95% CI: 1.1 -1.9) as well as intrauterine fetal death by 4.4 times (95% CI: 2.8-6.7) in populations of low socioeconomic status.^(16)^ In this population, poverty itself is a risk factor for SGA at 3,400-m altitude (OR=3.5; 95%CI, 2.4–5.1)^(17)^and at sea level.^(18)^

In low socioeconomic populations, FG^(19)^ and BW^(20)^ showed no significant statistical differences from the first trimester to 35 weeks of gestation at sea level and altitudes >3,000m. However, it changes after 36 weeks of gestation. Fetal biometry ≥ 36 weeks at 4,340m altitude shows 21% of these measurements <5th percentile of fetuses at sea level.^(19)^ BW in Cusco is 3,096±459 g compared to the BW (3,263±484 g) in Lima (150 m).^(20)^In populations with better socioeconomic conditions, the BW is higher. For example, the BW in Lima is 3,383±434 g, in Cusco is 3,262±393 g, and in Puno (3,840-m altitude) is 3,273±407 g. Likewise, neonatal anthropometric measurements in Cusco are similar to those described by the INTERGROWTH 21 Consortium.^(21)^

The adapted population to altitude presents changes at the level of different genes that allow an increase in Hb levels, red blood cells, nitric oxide, increased vasodilation, and blood flow, among other actions. All these actions allow erythrocytes to have a greater capacity for deformation, transport, and release oxygen to the tissues in the microcirculation.^(22)^ Tibetan women have experienced this process better^(8)^ than Andean women, who show a marked and slight decrease in Hb in the second and third trimesters of pregnancy,^(16)^ the stage in which the highest FG occurs. Other significant changes are a greater release of oxygen to the tissues and dilation of the blood vessels, especially the uterine artery, which allows greater blood flow despite the increase in viscosity.^(7)^ Opposite changes occur in pathological conditions such as PE, where the ME, <PN, and a greater possibility of fetal death are observed.^(23)^

To determine SGA in this study, we used TANA, developed in Cusco.^(12)^ Other researchers, such as Gonzales et al.,^(16)^ used the neonatal table developed by the Latin American Center for Perinatology and Human Development to evaluate newborns at high altitudes, which generated different results. This table was created in public hospitals in cities located <800 m altitude.^(24)^ Similarly, Ticona-Rendón and Huanco-Apaza^(25)^ determined a prevalence of 14.6% of SGA and a high risk of neonatal mortality (ORc=15.6; 95% CI, 8.4-28.9) in a population of low socioeconomic level in Cusco. To do this, they used a neonatal table developed in public hospitals in Peru, where the majority of the population studied (~70%) came from sea-level cities. Meanwhile, TANA has identified a prevalence of SGA of 9.6% and a lower risk of neonatal mortality (ORa=2.43; 95%CI, 1.03-6.51)^(26)^ in a middle-class population.

Inadequate prenatal control at 3,400-m altitude increases the risk of SGA (ORa=2.14; CI95%, 1.16-3.94 and p=0.015) compared to adequate prenatal control. At sea level, it has a protective effect for SGA (ORa=0.7; CI95%,0.67-0.73).^(27)^ Therefore, a greater number of controls allows better recognition of different factors that could be corrected and managed before they negatively impact on FG.

A limitation of this study was the lack of fetal Doppler ultrasound information to better discriminate the presence of intrauterine growth restriction.

## Conclusion

The maternal erythrocytosis in pregnant women without concomitant morbidity does not appear to be a risk factor for SGA at 3,400 m-altitude. Additional studies with a larger population of pregnant women are needed.

## References

[1] Gonzales GF (2012). Mother's hemoglobin in perinatal and mother health in the highlands: implications in the Andean Region. Rev Peru Med Exp Salud Publica.

[2] Gonzales GF, Tapia V, Gasco M, Carrillo CE, Fort AL (2012). Association of hemoglobin values at booking with adverse maternal outcomes among Peruvian populations living at different altitudes. Int J Gynaecol Obstet.

[3] Tremblay JC, Ainslie PN (2021). Global and country-level estimates of human population at high altitude. Proc Natl Acad Sci U S A.

[4] American College of Obstetrician and Gynecologists (2021). Fetal growth restriction: ACOG Practice Bulletin, Number 227. Obstet Gynecol.

[5] Knop MR, Geng TT, Gorny AW, Ding R, Li C, Ley SH (2018). Birth weight and risk of type 2 diabetes mellitus, cardiovascular disease, and hypertension in adults: a meta-analysis of 7 646 267 participants from 135 studies. J Am Heart Assoc.

[6] Julian CG, Vargas E, Armaza JF, Wilson MJ, Niermeyer S, Moore LG (2007). High-altitude ancestry protects against hypoxia-associated reductions in fetal growth. Arch Dis Child Fetal Neonatal Ed.

[7] Bigham AW, Lee FS (2014). Human high-altitude adaptation: forward genetics meets the HIF pathway. Genes Dev.

[8] Moore LG, Zamudio S, Zhuang J, Sun S, Droma T (2001). Oxygen transport in tibetan women during pregnancy at 3,658 m.. Am J Phys Anthropol.

[9] Villamonte W, Malaver J, Salinas R, Quispe E, Laurent A, Jeri M (2011). Conditioning parental factors for term birth weight at 3 400 m above sea level. Rev Peru Ginecol Obstet.

[10] Seguro Social de Salud (2015). Presentation of the main results of the national socio-economic survey of access to ESSALUD insured health.

[11] Gonzales GF, Tapia V, Fort AL (2012). Maternal and perinatal outcomes in second hemoglobin measurement in nonanemic women at first booking: effect of altitude of residence in Peru. ISRN Obstet Gynecol.

[12] Villamonte-Calanche W, Yabar-Galdos G, Jeri-Palomino M, Wilson NA (2019). Anthropometric reference curves for term neonates born at 3400 meters above sea level. J Matern Fetal Neonatal Med.

[13] Instituto Nacional de Estadística e Informática (2020). Map of provincial and district monetary poverty 2018.

[14] Julian CG, Moore LG (2019). Human genetic adaptation to high altitude: evidence from the Andes. Genes (Basel).

[15] Ministerio de Salud (2013). Technical health standard for comprehensive maternal health care.

[16] Gonzales GF, Steenland K, Tapia V (2009). Maternal hemoglobin level and fetal outcome at low and high altitudes. Am J Physiol Regul Integr Comp Physiol.

[17] Villamonte-Calanche W, Pereira-Victorio CJ, Jerí-Palomino M (2017). Anthropometry in at-term neonates in a rural and an urban population at 3 400 meters altitude. Rev Panam Salud Publica.

[18] Falcão IR, Ribeiro-Silva RC, de Almeida MF, Fiaccone RL, Silva NJ, Paixao ES (2021). Factors associated with small- and large-for-gestational-age in socioeconomically vulnerable individuals in the 100 Million Brazilian Cohort. Am J Clin Nutr.

[19] Krampl E, Lees C, Bland JM, Espinoza Dorado J, Moscoso G, Campbell S (2000). Fetal biometry at 4300 m compared to sea level in Peru. Ultrasound Obstet Gynecol.

[20] Hartinger S, Tapia V, Carrillo C, Bejarano L, Gonzales GF (2006). Birth weight at high altitudes in Peru. Int J Gynaecol Obstet.

[21] Villamonte-Calanche W, Manrique-Corazao F, Jerí-Palomino M, De-La-Torre C, Roque-Roque JS, Wilson NA (2017). Neonatal anthropometry at 3400 m above sea level compared with INTERGROWTH 21st standards. J Matern Fetal Neonatal Med.

[22] Zhao Y, Wang X, Noviana M, Hou M (2018). Nitric oxide in red blood cell adaptation to hypoxia. Acta Biochim Biophys Sin (Shanghai).

[23] Stephansson O, Dickman PW, Johansson A, Cnattingius S (2000). Maternal hemoglobin concentration during pregnancy and risk of stillbirth. JAMA.

[24] Fescina RH, De Mucio B, Martínez G, Alemán A, Sosa C, Mainero L (2013). Vigilancia del crecimiento fetal: manual de autoinstrucción.

[25] Ticona-Rendón M, Huanco-Apaza D (2007). Peruvian reference curves for birth weight according to gestational age and their application for identification new neonatal population with high risk. Rev Peru Med Exp Salud Publica.

[26] Villamonte-Calanche W (2022). Response to letter to the editor: methodological considerations on the article "Small and large for gestational age as a risk factor for neonatal morbidity and mortality at high altitude". Rev Cuerpo Med Hosp Nac Almanzor Aguinaga Asenjo.

[27] Suárez-Idueta L, Bedford H, Ohuma EO, Cortina-Borja M (2021). Maternal risk factors for small-for-gestational-age newborns in Mexico: analysis of a Nationwide Representative Cohort. Front Public Health.

